# Can inhaled cannabis users accurately evaluate impaired driving ability? A randomized controlled trial

**DOI:** 10.3389/fpubh.2023.1234765

**Published:** 2023-11-22

**Authors:** Sarah Hartley, Nicolas Simon, Bibiana Cardozo, Islam Amine Larabi, Jean Claude Alvarez

**Affiliations:** ^1^Sleep Unit, Physiology Department, AP-HP GHU Paris-Saclay, Raymond Poincaré Hospital, Garches, France; ^2^Department of Clinical Pharmacology, Aix Marseille Univ, APHM, INSERM, IRD, SESSTIM, Hop Sainte Marguerite, CAP-TV, Marseille, France; ^3^Plateform MasSpecLab, Department of Pharmacology and Toxicology, Raymond Poincaré Hospital, GHU AP-HP.Paris-Saclay, Paris-Saclay University, UVSQ, Inserm U-1018, CESP, Team MOODS, Garches, France

**Keywords:** cannabis, driving, driving simulator, reaction time, accident

## Abstract

**Aims:**

To study the effect of inhaled cannabis on self-assessed predicted driving ability and its relation to reaction times and driving ability on a driving simulator.

**Participants and methods:**

30 healthy male volunteers aged 18–34: 15 chronic (1–2 joints /day) and 15 occasional (1–2 joints/week) consumers. Self-assessed driving confidence (visual analog scale), vigilance (Karolinska), reaction time (mean reciprocal reaction time mRRT, psychomotor vigilance test), driving ability (standard deviation of lane position SDLP on a York driving simulator) and blood concentrations of delta-9-tétrahydrocannabinol (THC) were measured before and repeatedly after controlled inhalation of placebo, 10 mg or 30 mg of THC mixed with tobacco in a cigarette.

**Results:**

Cannabis consumption (at 10 and 30 mg) led to a marked decrease in driving confidence over the first 2 h which remained below baseline at 8 h. Driving confidence was related to THC dose and to THC concentrations in the effective compartment with a low concentration of 0.11 ng/ml for the EC50 and a rapid onset of action (T1/2 37 min). Driving ability and reaction times were reduced by cannabis consumption. Driving confidence was shown to be related to driving ability and reaction times in both chronic and occasional consumers.

**Conclusions:**

Cannabis consumption leads to a rapid reduction in driving confidence which is related to reduced ability on a driving simulator.

**Clinical trial registration:**

ClinicalTrials.gov, identifier: NCT02061020.

## 1 Introduction

Cannabis is the most widely used illicit drug in the world, and is also the most common illicit drug found in roadside testing: 29.8–36.9% of drivers driving under the influence of drugs (DUID) are found to have used cannabis (1, 2). The prevalence of DIUD seems to be increasing over time possibly linked to the progressive legalization of medical and recreational cannabis (3). Cannabis affects psychomotor and cognitive function (4), leading to increased reaction times (5). Driving is a complex task so the finding that driving under the influence of cannabis (DUIC) is linked with increased accidents (6) is not surprising. The increased risk has been estimated as an OR of 1.32 (95% CI = 1.09–1.59) (7), although as many studies rely on self-report this may underestimate the real prevalence. There is concern that the increase in the potency of recreationally consumed cannabis is increasing: a Norwegian study showed that the amount of THC had increased by 58% potentially increasing the impact on driving (8).

We have shown that inhaled cannabis has complex pharmacokinetics (9). The amount of THC ingested is dependent on inhalation techniques and as THC is lipophilic it can persist in fatty tissue, including the brain. Blood THC and oral fluid levels may thus not be directly proportional to impairment. In contrast, ethanol is highly water soluble and thus rapidly diffuses after consumption although this may be complicated by gastric emptying. Metabolism is rapid and breath alcohol concentrations (BAC) can be easily measured and are proportional to the ingested dose. Comparing the accident risk of alcohol and cannabis is thus complicated by issues of measurement (10).

Alcohol remains a major risk factor for accidents, but the percentage of drivers involved in fatal accidents who have used alcohol has remained stable over time (11). The risk of accident associated with the use of alcohol is high: a threefold increase in accident risk is seen with a blood alcohol concentration (BAC) of 0.01 – 0.05 and a thirtyfold increase with a BAC of 0.08–0.12 (12). A study of fatal accidents using drivers testing negative for both alcohol and cannabis as the reference found an adjusted OR of 16.33 [95% CI: 14.23, 18.75] for alcohol alone, 1.54 (95% CI: 1.16, 2.03) for cannabis alone and 25.09 (95% CI: 17.97, 35.03) alcohol and cannabis (13). In simulator studies, both alcohol and cannabis affect driving ability (14–17) but their effects, while dose dependant, are similar: Hartman found a 16% increase in the standard deviation of lateral position (SDLP) with 20 μg/L of THC vs. a 13% increase with 0.1 g/210 L breath alcohol concentration (18).

It has been suggested that the effects on driving ability in cannabis consumers may be limited to a degree by reduced speed and increased headway (17, 18). This may partially compensate for reduced ability (19). However, the adoption of compensatory measures requires the perception that ability is reduced. A recent study performed without blood THC levels found that 50% of young occasional cannabis users thought their driving was unaffected after the inhalation of 13 mg of THC, although they thought they were less safe to drive than if they had not smoked cannabis (20). Arkell showed that both driving confidence and driving ability measured by driving simulator are reduced by THC with a persistent effect at 240 min, after the resolution of objectified cognitive impairment and psychological symptoms (15). The precise relationship between driving confidence, driving ability, and pharmacokinetically modeled THC levels has not been studied.

The primary objective of the present study was to examine the relationship between self-evaluated driving confidence, driving ability measured on a driving simulator, reaction time, and the dose and concentration of THC.

## 2 Methods

The study was approved by the local ethics committee and conducted in compliance with good clinical practice guidelines and the Declaration of Helsinki. All participants provided written informed consent.

### 2.1 Population

Thirty healthy male volunteers aged 20–34 who had consumed cannabis for at least 1 year and held a valid driving license were recruited. The participants were divided into two groups: occasional consumers (OC) consuming 1–2 joints per week and chronic consumers (CC) consuming 1–2 joints per day. Exclusion criteria included excessive alcohol use (AUDIT score >13), usual daily intake exceeding 225 mg caffeine per day, symptoms compatible with medical, psychiatric, or sleep disorders, and consumption of drugs other than cannabis or psychoactive medication in the month prior to inclusion. Initial exclusion of consumption of other drugs relied on self-report, but this was confirmed by urine analysis using liquid chromatography coupled to mass spectrometry (psychotropic medications such as antidepressants, anxiolytics, hypnotics, and narcotic use other than cannabis i.e., opiates, cocaine, and amphetamines). Cannabis use was confirmed by urinary drug screening (using liquid chromatography and mass spectrometry) and chronic use was confirmed by capillary analysis.

### 2.2 Study design and procedures

This was a pilot, randomized, blinded, cross-over study. Each participant underwent three 24-h sessions in random order, with a 7-day wash-out between each session. At the inclusion visit a sleep physician and a psychiatrist saw all participants to check the inclusion and exclusion criteria. The sleep physician interview aimed to exclude sleep pathology (e.g., sleep apnea) associated with reduced vigilance and examined sleep behaviors in order to exclude circadian rhythm disorders, in particular delayed sleep phase disorder, which affects diurnal vigilance. The psychiatrist interview excluded psychiatric disorders complicating cannabis use and administered the AUDIT questionnaire to screen for excessive alcohol use. Participants were encouraged to consider withdrawal from cannabis use at the end of the study and referred to appropriate services. All participants underwent an ECG and were trained on the psychomotor vigilance test (PVT) and driving simulator.

Each of the three sessions started at 08:00 and lasted 24 h. No cannabis use was allowed from 12:00 the day before and all participants underwent an oral fluid drug test on arrival to confirm abstention. A venous line for plasma THC sampling was inserted. Baseline questionnaires, 30-min simulated driving, and PVT were performed. This was followed by a 10-min protocolized cannabis inhalation using a cannabis-containing cigarette with monitoring of heart rate, blood pressure, and respiratory rate. Expired carbon monoxide levels were monitored before and after consumption. Blood samples were taken before consumption (for baseline THC concentration and measurement of alcohol), 5 min, 15 min, 30 min, 1 h, 2 h, 4 h, 6 h, 8 h, 10 h, 12 h, and 24 h after the end of the cigarette. Questionnaires, PVT, and driving simulator in randomized order for each participant were performed at 1 h, 2 h, 4 h, 6 h, 8 h, 12 h, and 24 h.

### 2.3 Interventions

Cannabis in leaf form from the regional police department was powdered and THC concentration was measured by liquid chromatography and mass spectrometry. Textile-grade hemp was used as a placebo. Cannabis-containing cigarettes were prepared using strongly perfumed tobacco (Amsterdamer^®^) to hide the characteristic aroma of cannabis. Over the three sessions, each participant received cigarettes containing 1 g tobacco and either placebo, 10 mg, or 30 mg of THC. The order of cigarettes was randomized using a computer-generated algorithm by the Clinical Research Unit of the Hospital for each participant and cigarettes were then prepared by a non-participant member of the laboratory staff. Both participants and investigators were blinded to the content of cigarettes. The inhalation protocol was as follows: inhalation for 2 s every 20 s over a period of 10 min. Residual material from the cigarette was recovered and analyzed to determine the total amount of THC consumed. The amount of inhalation was determined by analysis of exhaled carbon monoxide.

### 2.4 Testing and outcome measures

#### 2.4.1 Questionnaires

Participants were asked to rate their confidence in their ability to drive safely on a 100-mm visual analog scale: from not at all confident (0) to very confident (100), before each series of tests. Results of driving confidence were expressed as %. Subjective vigilance was evaluated using the Karolinska Sleepiness scale which measures vigilance on a 9-point scale (1 = extremely alert, to 9 = extremely sleepy, can't keep awake) (21).

#### 2.4.2 Driving simulation

Each 30-min period of driving simulation was performed using a York driving simulator (York Computer Technologies, Ontario, Canada). The task was monotonous with a simulated four-lane road, speed limits in km/hour which the drivers had to follow, simulated gusts of wind causing deviation of the trajectory needing lane position correction, and occasional passing cars (22). The outcome measure was driving ability expressed as the standard deviation from the central road position (SDLP in percentile).

#### 2.4.3 Vigilance testing

Objective vigilance was assessed using 10-min PVT (23). The outcome measure was reaction time expressed as the mean reciprocal reaction time (mRRT or 1/mean reaction time in seconds).

#### 2.4.4 Blood testing

THC, 11-OH-THC, and THC-COOH were measured by liquid chromatography and mass spectrometry allowing the detection of THC and 11-OH-THC down to 0, 2 ng/mL and THC-COOH to 1 ng/mL. All tests were performed in our nationally accredited toxicology laboratory.

### 2.5 Statistical analysis

The relationship between driving confidence (CDA) and driving ability (SDLP), reaction time (mRRT), and sleepiness (Karolinska sleepiness scale) were analyzed using the lme4 package (24) with the R program (25) which enables to perform statistical analyses for repeated measures. The association between CDA and SDLP, mRRT, sleepiness, THC dose (mg), group (occasional vs. chronic user), and time (h) was evaluated by linear regression analyses using the lmer function with the patient as a grouping factor to estimate the random-effects term.

#### 2.5.1 Population PK/PD analysis

Population pharmacokinetic/pharmacodynamic (PK/PD) analysis using a non-linear mixed effect modeling in NONMEM version 7.4.1 (26) with the gfortran 4.6.0 compiler was performed. Wings for NONMEM version 743 was used as a “front end” for the NONMEM program [Holford]. Graphical analysis used the R software version 3.4.2 [R] and the ggplot2 package. The first-order conditional estimate (FOCE) method with the interaction option was used. This pharmacokinetic approach has already been published and was used to identify the PK/PD model (9). A sequential approach, as defined by Zhang et al. (27), was applied to identify the best model and to estimate the PD parameters. The outcome was the confidence in driving ability (CDA) based on a 100-mm visual analog scale. Several PK/PD models such as linear, log-linear, simple Emax, and sigmoid were tested using a direct model or an effect compartment implemented with ADVAN6 and differential equations. The following pharmacodynamic parameters were used to parameterize the models: KE0 (effect-site equilibration rate constant for CDA), EC50 (concentration leading to 50% of the maximum CDA effect), E0 (effect at time 0 for CDA), and Emax (maximum effect for CDA). An exponential error model was used to estimate the between-subject variability (BSV) of the pharmacodynamic parameters. Residual unexplained variability (additive, proportional, and mixed) was assessed by several error models. An effect of the group (chronic vs. occasional) was evaluated on each parameter. Model performance was judged by both graphic and statistical methods (28). Goodness-of-fit was assessed by the minimal value of the objective function: we note that increased goodness-of-fit is accompanied by decreased objective function value, which is asymptotically distributed as a chi-square distribution. The COVARIANCE option in NONMEM was used to calculate standard errors. The following diagnostic plots were evaluated: observed concentrations [dependent variable (DV)] vs. PRED, individual predictions (IPRED) vs. DV, conditional weighted residuals (cWRES) vs. time and cWRES vs. PRED. parameter estimate precision was expressed as Relative Standard Error (RSE, %) and confidence intervals (CI). NONMEM was used to directly calculate the RSE with an acceptable value <30% for fixed effects and <50% for random effects. The robustness of standard approximations for parameter uncertainty were verified by a bootstrap method using the lower 2.5% and the upper 97.5% value of each parameter percentile as the bootstrap confidence intervals.

## 3 Results

### 3.1 Participants

**Figure 1:**
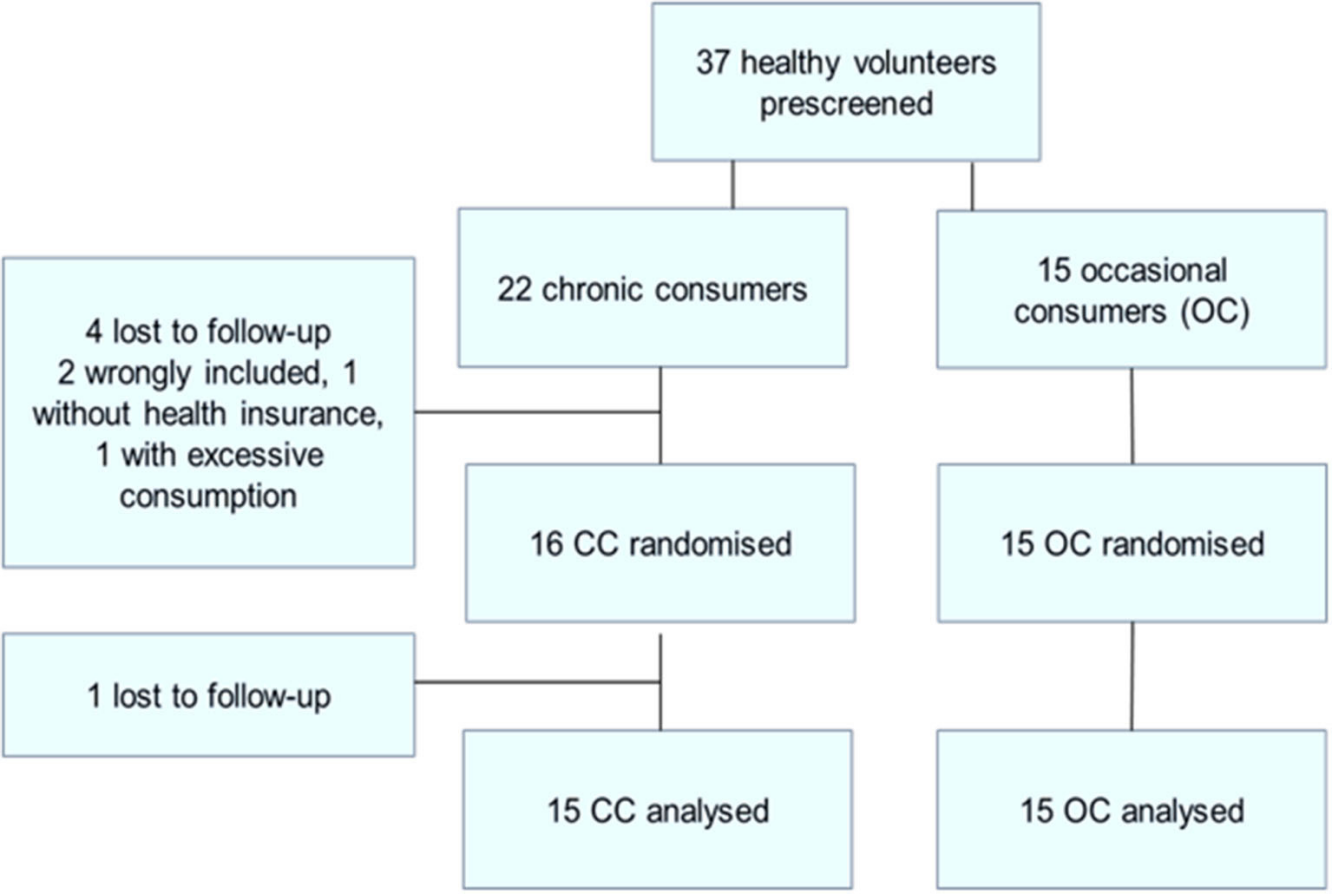
Flow chart.

A total of 37 healthy male cannabis smokers were pre-screened (see Figure 1: flowchart of inclusions) of whom 30 completed the study. Fifteen participants completed the study in each of the two groups [occasional cannabis consumers (OC) and chronic cannabis consumers (CC)] in the CC group 22 were included: 4 were lost to follow-up, one did not have health insurance and one had excessive THC use (>2 cigarette/day).

There was no significant difference between the groups for age, BMI, daytime sleepiness, chronotype as measured by the Horne and Ostberg score or educational level. All drank alcohol but tobacco (except in cannabis-containing cigarettes) was not found in all participants. Cigarette and alcohol consumption (Audit score), and coffee consumption (cups per day) were not significantly different between the two groups.

### 3.2 Driving confidence

**Figure 2:**
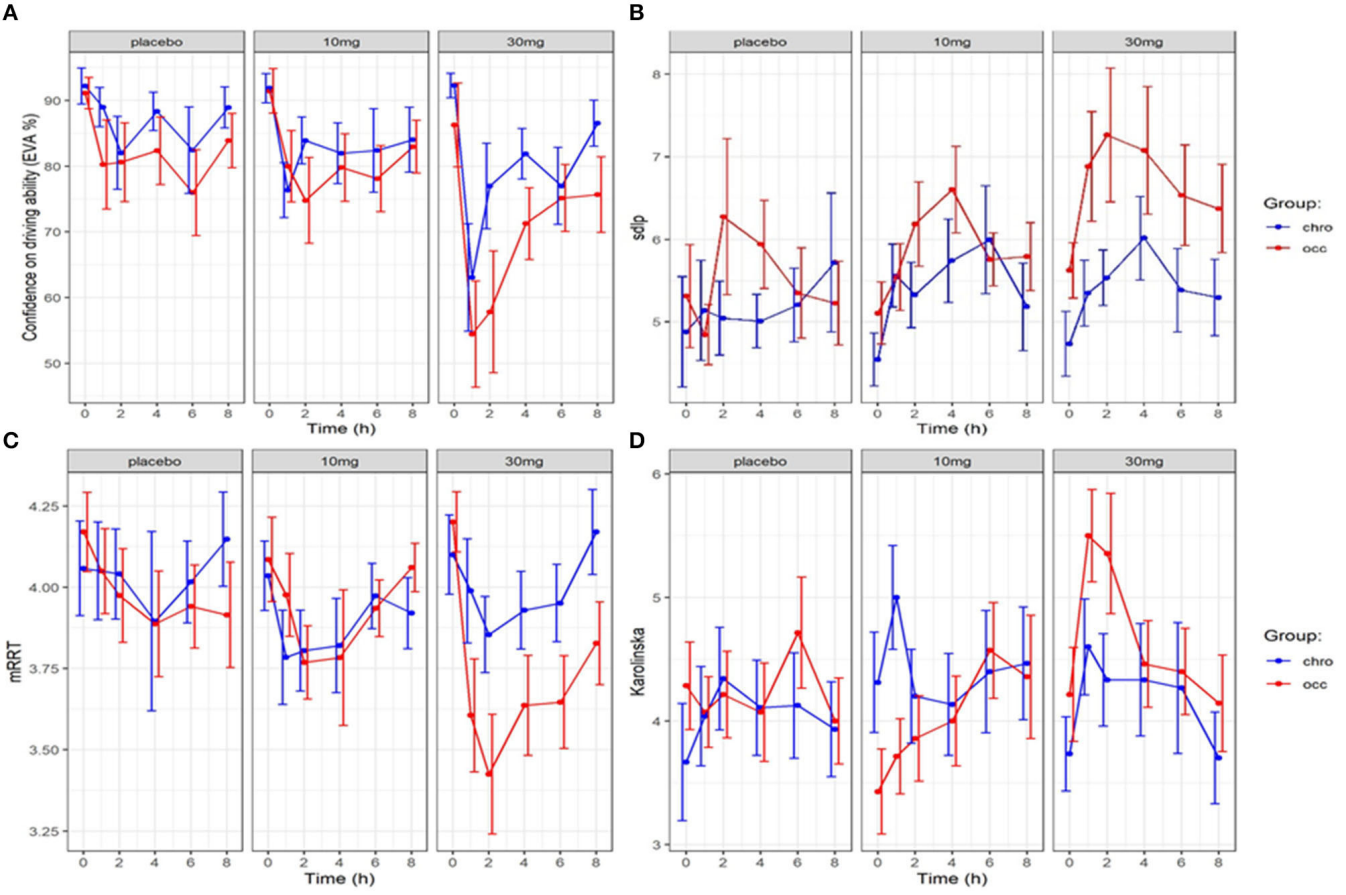
Evolution in confidence in driving, driving ability (SDLP), reaction time (mRRT) and sleepiness (Karolinska sleepiness scale): effect of time and dose of THC (mean +/–SEM). **(A)** Driving confidence (EVA) over time, **(B)** Driving ability (SDLP) over time), **(C)** reaction time (mRRT) over time, **(D)** Sleepiness (Karolinska) over time.

**Figure 3:**
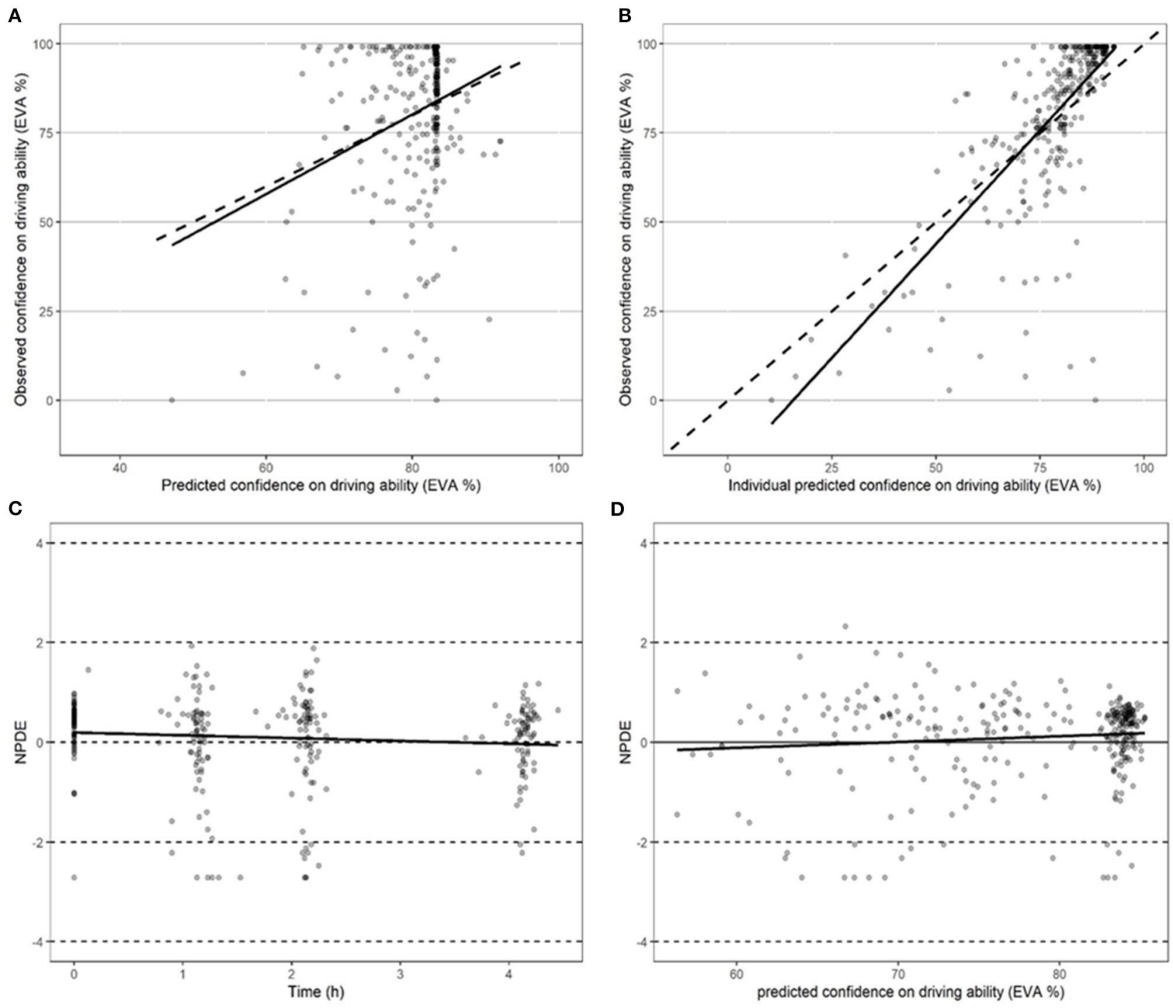
Performance of models in predicting driving confidence. **(A)** Performance of the model in predicting driving confidence using actual population parameters. **(B)** Performance of the model in predicting driving confidence based on optimized Bayesian parameters. **(C)** Performance of the model in predicting normalized predictive distribution error (NPDE) over time points. **(D)** Performance of the model in predicting normalized predictive distribution error (NPDE) vs. driving confidence.

The evolution over time in driving confidence is shown in Figure 2. Data is reported over 8 h, the period during which impairment was detected in driving ability. Driving confidence decreases markedly after the consumption of cannabis, reaching a nadir at 1 hour after the consumption of 30 mg, followed by a progressive increase. No difference was found between groups. We fitted a linear mixed model (estimated using REML and nloptwrap optimizer) to predict RESS with GRP [formula: RESS ~ GRP + DOSE + factor (ttime)]. The model included ID as a random effect (formula: ~1 | ID). The model was fitted on a standardized version of the dataset to obtain standardized parameters. A total of 95% A Wald t-distribution approximation was used to calculate confidence Intervals (CIs) and *p*-values. The model's total explanatory power is substantial (conditional R^2^ = 0.46) and the part related to the fixed effects alone (marginal R^2^) is of 0.12. The model's intercept, corresponding to GRP = chro, is at 98.11 (95% CI [90.65, 105.56], *t*(503) = 25.86, *p* < 0.001), see Figure 3. In this model dose and time significantly and negatively affect confidence in driving but the group is not statistically significant (Table 1).

**Table 1:** Model of confidence in driving.

**Confidence in driving ability**							
		**Beta**	**95% CI**	***t*** **(503)**	***p***	**Std beta**	**95% CI**
Dose		−0.35	[−0.46, −0.24]	−6.19	< 0.001	−0.20	−0.27, −0.14
Group		−4.92	[−14.49, 4.66]	−1.01	0.314	−0.23	−0.68, 0.22
Time	1	−13.87	[−18.54, −9.19]	−5.83	< 0.001	−0.86	−1.09, −0.64
	2	−9.96	[−14.73, −5.19]	−4.10	< 0.001	−0.65	−0.88, −0.43
	4	−12.32	[−17.04, −7.61]	−5.13	< 0.001	−0.47	−0.69, −0.24
	6	−18.29	[−23.08, −13.50]	−7.50	< 0.001	−0.58	−0.80, −0.36
	8	−7.18	[−11.92, −2.44]	−2.98	0.003	−0.34	−0.56, −0.12

**Figure 4:**
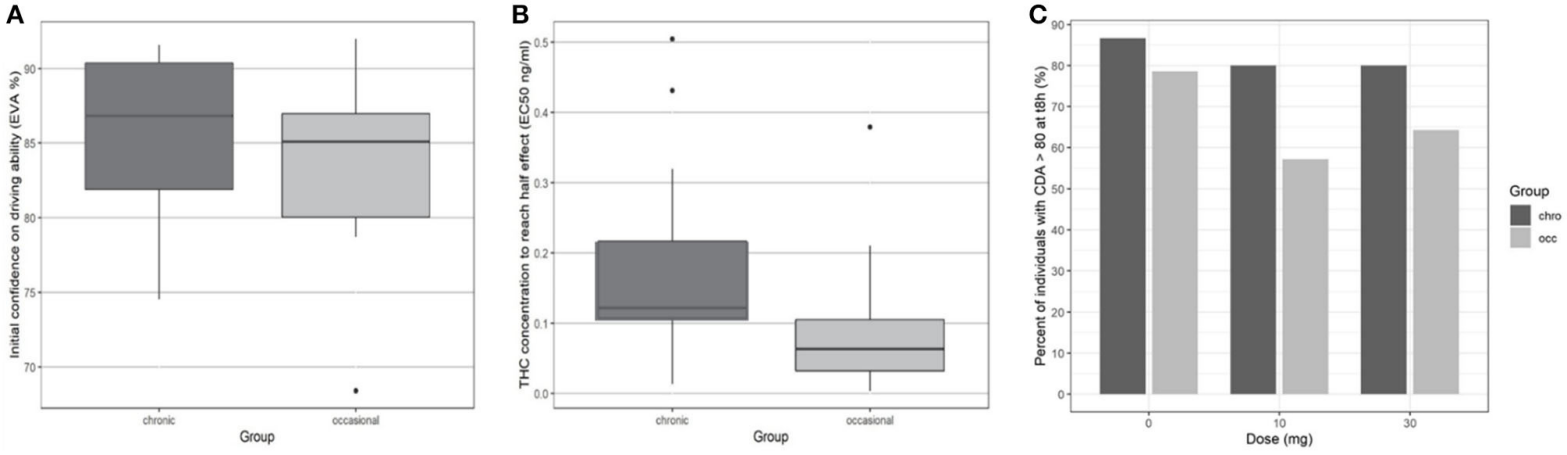
Driving confidence initially and according to THC concentration by group. **(A)** Initial driving confidence E0 by group. **(B)** Mean THC concentration to reach half effect EC50. **(C)** Percentage of participants who returned to at least 80% of their original confidence in driving ability at 8 h.

At the end of the 8-h period, driving confidence remained below baseline. We studied the number of participants according to the group who at 8 h had returned to at least 80% of their initial driving confidence (see Figure 4). No significant difference was found for either dose or group in the return of driving confidence.

### 3.3 Relation between driving confidence and driving ability (SDLP)

**Figure 5:**
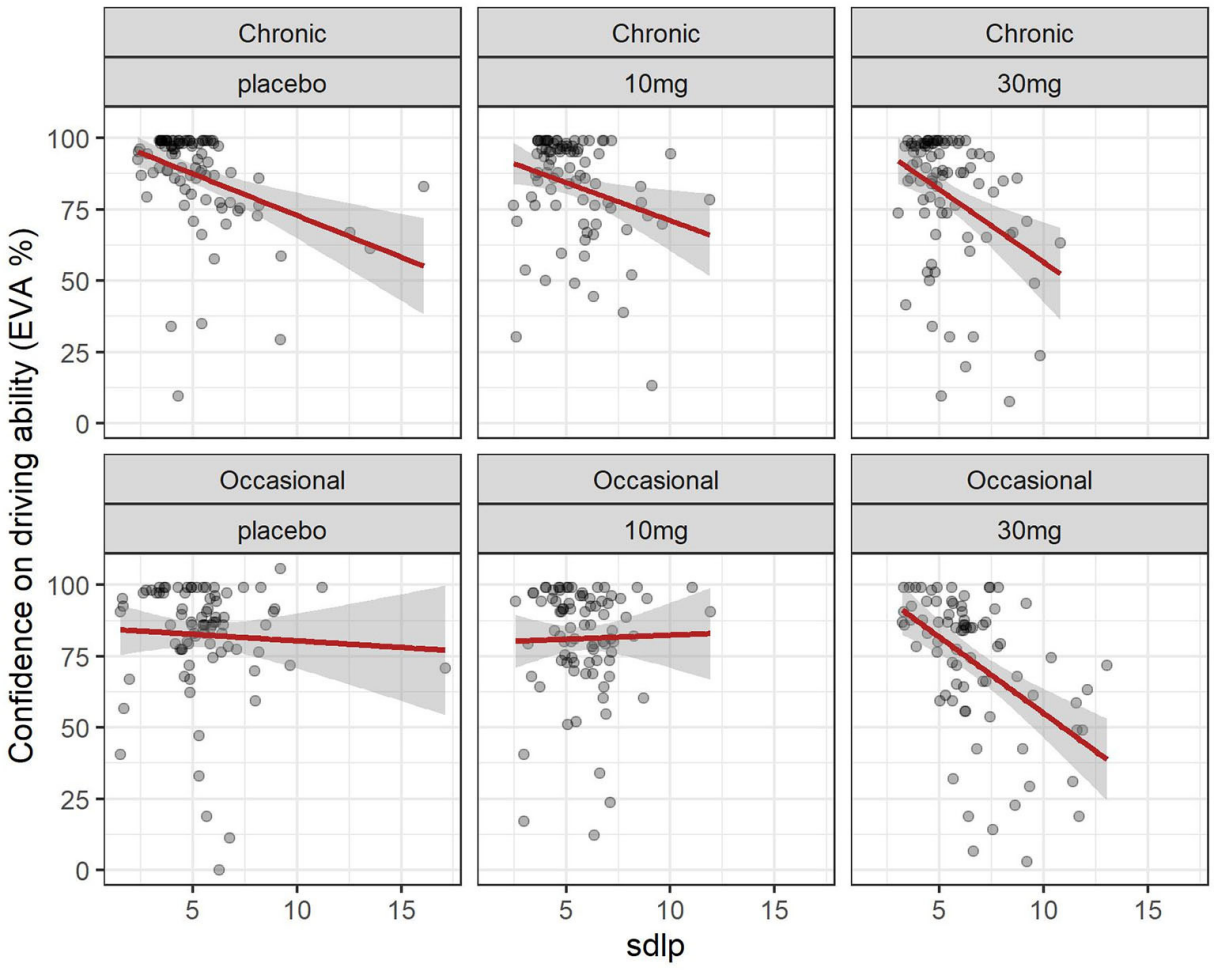
Driving confidence and driving ability (SDLP): effect of dose in chronic and occasional consumers. Relation between driving confidence and driving ability (SDLP) in occasional and chronic cannabis consumers according to dose. formula: EVA ~ sdlp + DOSE + GRP + factor (time).

The evolution of SDLP over time in the function of group and dose is shown in Figure 2. A linear mixed model (estimated using REML and nloptwrap optimizer) was fitted to predict confidence in driving ability from SDLP (Figure 5). The model was fitted on a standardized version of the dataset to obtain standardized parameters. A total of 95% A Wald t-distribution approximation was used to calculate confidence Intervals (CIs) and *p*-values. The model's total explanatory power is substantial (conditional R^2^ = 0.47) and the part related to the fixed effects alone (marginal R^2^) is 0.17. The model's intercept, corresponding to sdlp = 0, is at 108.80 (95% CI [100.75, 116.86], *t*(491) = 26.53, *p* < 0.001), see Table 2. Within this model the effect of the SDLP is statistically significant and negative, the effect of the dose of THC is statistically significant and negative but the effect of the group (occasional vs. regular consumers) is not significant.

**Table 2:** Model of confidence in driving and driving ability (SDLP), reaction time (mRRT) and daytime sleepinesss (Karolinska sleepiness scale).

**Confidence in driving and driving ability (SDLP)**							
		**Beta**	**95% CI**	**t (491)**	***p***	**Std beta**	**95% CI**
SDLP		−2.66	−3.58, −1.74	−5.67	< 0.001	−0.26	−0.35, −0.17
Dose		−0.24	−0.36, −0.13	−4.34	< 0.001	−0.15	−0.21, −0.08
Group		−3.10	−11.95, 5.75	−0.69	0.667	−0.15	−0.58, 0.28
Time (hours)	1	−15.50	−20.23, −10.77	−6.44	< 0.001	−0.75	−0.98, −0.52
	2	−11.42	−16.13, −6.71	−4.76	< 0.001	−0.55	−0.78, −0.33
	4	−6.25	−11.03, −1.47	−2.57	0.011	−0.30	−0.54, −0.07
	6	−9.89	−14.57, −5.20	−4.15	< 0.001	−0.48	−0.71, −0.25
	8	−5.33	−10.01, −0.64	−2.23	0.026	−0.26	−0.49, −0.03
**Confidence in driving and reaction time (mRRT)**							
		**Beta**	**95% CI**	**t (502)**	***p***	**Std beta**	**95% CI**
mRRT		9.33	5.48, 13.18	4.76	< 0 0.001	0.23	0.13, 0.32
Dose		−0.30	[−0.41, −0.19]	−5.34	< 0.001	−0.18	−0.24, −0.11
Group		−3.87	−13.39, 5.65	−0.69	0.425	−0.18	−0.63, 0.27
Time (hours)	1	−16.53	−21.28, −11.79	−6.85	< 0 0.001	−0.78	−1.00, −0.56
	2	−11.33	[−16.02, −6.64]	−4.74	< 0 0.001	−0.53	−0.76, −0.31
	4	−7.99	−12.73, −3.25	−3.31	< 0 0.001	−0.38	−0.60, −0.15
	6	−10.46	−15.14, −5.78	−4.39	< 0.001	−0.49	−0.71, −0.27
	8	−6.29	−10.94, −1.64	2.66	0.008	−0.30	−0.52, −0.08
**Confidence in driving and daytime sleepinesss (Karolinska sleepiness scale)**							
		**Beta**	**95% CI**	**t (517)**	***p***	**Std beta**	**95% CI**
KSS		−6.35	−7.38, −5.32	−12.11	< 0.001	−0.46	−0.53, −0.39
Dose		−0.26	−0.36, −0.16	−5.18	< 0 0.001	−0.15	−0.21, 0.10
Group		−4.46	−11.48, 2.21	−1.33	0.184	−0.22	−0.54, 0.10
Time	1	−14.22	−18.56, −9.88	−6.43	< 0 0.001	−0.67	−0.88, −0.47
	2	−12.64	−16.87, −8.40	−5.86	< 0.001	−0.60	−0.80, −0.40
	4	−8.74	−13.02, −4.45	−4.01	< 0.001	−0.41	−0.61, −0.21
	6	−9.41	−13.64, −5.18	−4.37	< 0.001	−0.44	−0.64, −0.24
	8	−6.63	−10.88, −2.37	−3.06	0.002	−0.31	−0.51, −0.11

Overall driving confidence reflects actual driving ability measured by the SDLP (see Figure 5), and is affected by the dose of THC: at a dose of 30 mg markedly reduced confidence reflects the reduction in driving ability. The relationship between confidence and ability seems less marked for occasional users at placebo or 10 mg levels but there is a large variation. In chronic users, there is a closer relationship between driving confidence and driving ability.

### 3.4 Relation between driving confidence and reaction times (mRRT)

**Figure 6:**
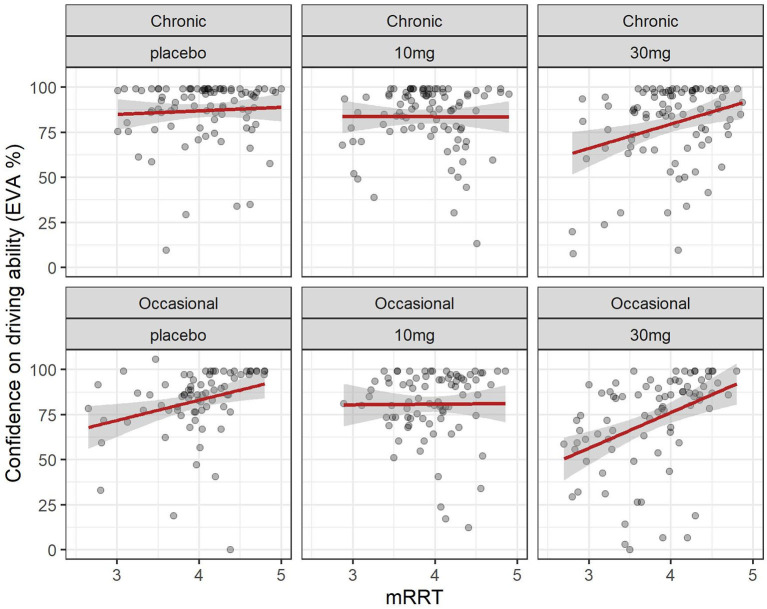
Driving confidence and reaction time (mRRT): effect of dose in chronic and occasional consumers. Relation between driving confidence and reaction time (mRRT) in occasional and chronic cannabis. consumers according to dose. Formula: EVA ~ mRRT + GRP + DOSE + factor (time).

The evolution of mRRT over time in function of the group and dose is shown in Figure 2. A linear mixed model (estimated using REML and nloptwrap optimizer) was fitted to predict confidence in driving ability from mRRT (see Figure 6). The model included ID as a random effect (formula: ~1 | ID). The model was fitted on a standardized version of the dataset to obtain standardized parameters. A total of 95% A Wald t-distribution approximation was used to calculate confidence Intervals (CIs) and *p*-values. The model's total explanatory power is substantial (conditional R^2^ = 0.50) and the part related to the fixed effects alone (marginal R^2^) is 0.17. The model's intercept, corresponding to mRRT = 0, is at 58.66 (95% CI [40.78, 76.54], *t*(502) = 6.45, *p* < 0.001) (see Table 2).

Overall confidence in driving is associated with reaction time (see Figure 6): increased driving confidence is associated with an increase in mRRT and thus a reduction in reaction time. This is not affected by group but is affected by dose: increasing dose leads to a reduction in mRRT (and thus an increase in reaction time).

### 3.5 Population PK/PD analysis of driving confidence

The relationship between THC and driving confidence (DC) was best described by an indirect (delayed) effect compartment model and a sigmoid equation (Eq. 1) with a maximum effect set to 1.


(1) $$\begin{array}{l} \text{DC}=\text{E}0{\;}^{*}\;\left(1-\text{Ce}/\left(\text{EC}50+\text{Ce}\right)\right) \end{array}$$

In Eq. 1, EC50 represents the concentration leading to 50 % of the maximum effect of DC, E0 the value of DC at time 0, and Ce the concentration in the biophase. The pharmacodynamic parameters, the residual standard error, and the bootstrap are presented in Table 3. Inter-individual variability was evaluated for E0 and EC50 and residual variability was modeled as proportional. A group effect was assessed on the PD parameters but was not significant and did not improve the fit. The diagnostic plots did not depict any bias and the bootstrap confirmed the robustness of the parameters' estimation. As can be seen, on average an extremely small amount of THC in the biophase compartment (EC50 = 0.11 ng/ml) is sufficient to affect driving confidence. The concentration of THC in the effective compartment required to reach EC50 seems lower for occasional consumers (Figure 6), but this is not significant due to relatively high interindividual variation, and we note that the concentration of THC in the effective compartment in this group is below the limit of quantification. Onset is rapid and expressed by the relationship T1/2 log (2)/1.13 = 0.61h (37 min).

**Table 3:** Pharmacodynamic parameters and driving confidence.

**Parameters**	**Unit**	**Value**	**RSE (%)**	**Bootstrap CI90**		
				**0.025**	**0.500**	**0.975**
Ke0		1.13	35.4	0.35	1.25	4.1
E0		83.4	3.3	76.8	83.8	88.6
EC50	(ng/mL)	0.11	39.6	0.04	0.11	0.91
**Inter individual variability (**ω**)**						
EC50		1.58	17.1	0.001	1.500	2.920
E0		0.09	23.1	0.003	0.087	0.132
**Residual unexplained variability (**σ**)**						
Proportional		0.24	14.3	0.169	0.237	0.315

Ke0, constant transfer to the effect compartment; E0, initial EVA; EC50, THC concentration in the effect compartment with half maximal effect; RSE, residual standard error.

### 3.6 Driving confidence and sleepiness (Karolinska sleepiness scale)

**Figure 7:**
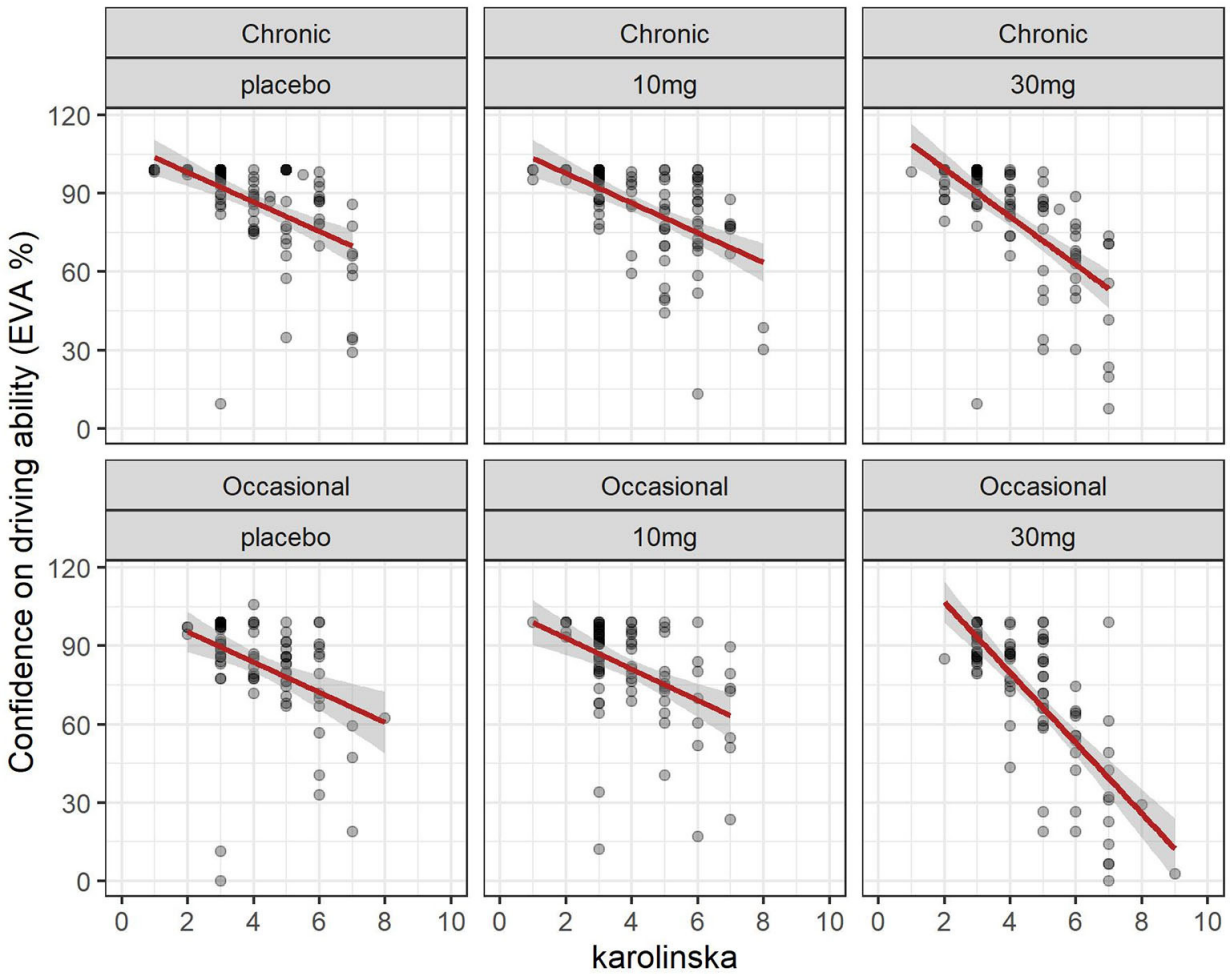
Driving confidence and sleepiness (Karolinska scale): effect of dose in chronic and occasional consumers.

The evolution of the Karolinska sleepiness score over time in function of the group and dose is shown in Figure 2. At a dose of 30 mg, sleepiness rises steeply in both groups to a maximum of 90 min before slowly falling back. There is a close relationship between sleepiness and driv ing confidence. We fitted a linear mixed model (estimated using REML and nloptwrap optimizer) to predict RESS with karolinska [formula: EVA ~ karolinska + GRP + DOSE + factor (time)]. The model included ID as a random effect (formula: ~1 | ID). The model was fitted on a standardized version of the dataset to obtain standardized parameters. A total of 95% A Wald t-distribution approximation was used to calculate confidence Intervals (CIs) and *p*-values. The model's total explanatory power is substantial (conditional R^2^ = 0.52) and the part related to the fixed effects alone (marginal R^2^) is 0.34. The model's intercept, corresponding to Karolinska = 0, is at 122.13 (95% CI [115.30, 128.96], *t*(517) = 35.14, *p* < 0.001), see Table 2. The model shows a strong relationship between sleepiness and driving confidence which is related to dose but not to group and strongly related to time with a persistent effect at 8 h (Figure 7).

## 4 Discussion

Our results show that driving confidence rapidly reduces following the consumption of cannabis, and this is related to cannabis dose and THC concentration in the effective compartment. Driving confidence is related both to driving ability (SDLP) and reaction time (mRRT) and is strongly linked to subjective sleepiness.

We have shown in previous studies that reaction time measured by the mRRT and driving ability measured by the SDLP is clearly reduced in a dose-dependent relationship with THC concentration in the effective compartment. Cannabis directly affects brain function via its actions on endocannabinoid receptors: fMRI shows acute changes in brain activity in areas related to the performance of tasks, saliency detection, and self-oriented mental activity (29). Cannabis leads to sedation, altered perception, and euphoria (30) and decreases cognitive and psychomotor function (4). It thus affects several functions necessary for driving: increased reaction time affects speed adjustments, braking, and adaptions to lane position, reduced sustained and divided attention may lead to difficulties adapting to changing road conditions and finally distortion of time perception may lead to underestimating the time required for adaptive maneuvers (4, 10, 31). The effects of THC on performance are known to increase as tasks become more complex (14) and studies show that simple driving-related tasks are less affected than complex tasks (20).

The impact on driving ability has been shown experimentally using driving simulators which have been shown to correlate with highway driving (32). Cannabis leads to increased reaction time (5, 33) increased lane weaving (14–16), and increased risk-taking (34) partially compensated by an increase in caution (35). Hartman's study showed similar effects on SDLP at the highest doses of THC and alcohol (18) but noted that alcohol had additional effects on lateral acceleration and lane departures. In a systematic review and metanalysis of the effects of cannabis and alcohol, while most studies looked at the cumulative effects of cannabis and alcohol, Simmons et al. noted that on driving simulator studies comparing cannabis to alcohol at BAC >0.04%, alcohol has greater adverse effects on lane position, lane excursions and is associated with increased speed, while cannabis is associated with increased caution and decreased speed (36). These finding imply that cannabis consumers are aware to some degree of their reduced capacity: they have a reduction in driving confidence.

The preferred mode of cannabis consumption is via smoking, and reflecting the real-life effects of cannabis on driving requires a meticulously controlled protocol with timed consumption of smoked cannabis, and regular performance testing to evaluate driving ability. Several studies have shown that cannabis smokers are able to self-titrate the amount of THC inhaled (16, 18, 37). The resulting variability in blood THC means that THC concentration is a more accurate parameter than the dose of THC. THC then has to penetrate the brain to affect cognitive performance.

We have shown that driving confidence precipitously declines in the 2 h after consumption reflecting the rapid absorption of cannabis when inhaled. Our results confirm those of Marcotte who shows a similar sharp decline up to 90 min post consumption (38). Our pharmacodynamic modeling shows that only low concentrations of THC are necessary in the effective compartment to negatively affect driving confidence and that the onset is relatively rapid: the equilibration half-life, which describes the time course of equilibration between the plasma and the effective compartment, is on average 37 min. Users of cannabis thus have a relatively rapid onset of action after smoking as the drug rapidly penetrates the brain leading to both a reduction in driving ability mirrored by a reduction in driving confidence. This rapid reduction in driving confidence may not only influence the decision to drive, but also allow the driver to put in place countermeasures. Alcohol absorption depends on the type of alcohol consumed and the presence of food in the stomach, potentially prolonging the time to Cmax (39) meaning that a driver may have already taken the wheel before the effects of alcohol become apparent. A well-controlled study by Garrisson (40) shows a sharp dissociation between self-evaluation of driving ability and actual performance in conditions of both low and high levels of alcohol consumption (BAC of 0.07% and 0.04%) confirming the findings of Tiplady that alcohol leads to overestimation of performance (41). Drivers who have consumed alcohol are thus falsely confident about their driving ability, whereas drivers who have consumed cannabis have some insight into their impairment and can take countermeasures, for example deciding not to drive. This difference in driving confidence between alcohol and cannabis may explain the differences in accident rates despite the fact that both alcohol and cannabis negatively affect driving ability.

We show that after the initial sharp reduction in driving confidence, confidence gradually increased. However, we found that driving confidence remained affected even after 8 h; the last period in which we could demonstrate an effect of cannabis consumption on driving ability. This may be due to the close relationship between driving confidence and sleepiness (measured by the Karolinska scale). The initial period of cannabis consumption was at 9 am, a period where the circadian clock favors wakefulness in people with a normal sleep cycle. Circadian effects lead to increasing sleepiness over time (42) and sleepiness is known to negatively affect driving ability (43). We showed a close relationship between estimated driving ability and sleepiness in both occasional and chronic consumers. As sleepiness increases over the day, even though cannabis consumption no longer affects driving ability the expected normalization in driving confidence does not occur.

We note that our participants consumed cannabis at 9 am, and our participants were thus less sleepy than a cannabis consumer who typically consumes cannabis in the evening. We hypothesize that the effect of the circadian clock leading to sleepiness in the evening could increase the known effects of cannabis consumption on driving ability. However, it has been shown that in conditions of sleep deprivation insight into reduced performance is retained (44) and as driving confidence is linked to sleepiness it may be that this will further reduce driving confidence and encourage the adoption of appropriate countermeasures.

Several studies demonstrate that the effects on driving ability observed in occasional cannabis smokers are more marked than those observed in chronic smokers (16, 45–47). In our previous study, we showed that occasional consumers showed an equivalent reduction in driving ability measured by the SDLP at a lower estimated EC50 in the brain compartment, implying habituation to the effects of cannabis in chronic consumers. While both occasional and chronic cannabis consumers retain some insight into the effects of cannabis on their driving (with a reduction in both driving confidence and driving ability measured by the SDLP) chronic consumers seem to have a better estimation of potential impairment compared to occasional consumers under placebo and low dose (10 mg) cannabis. It is possible that this is due to the fact that our chronic consumers, who use cannabis daily, have direct experience of driving while under the influence of cannabis and thus are more able to evaluate their driving ability.

Even if cannabis users retain some insight into their impaired driving ability it is clear that their accident risk is increased. As a result, public health measures to reduce cannabis use are of interest. Sobriety checkpoint programs have been shown to be effective in limiting driving under the influence of alcohol (48) and there is evidence that this may be effective for cannabis (49). The method best adapted to roadside testing is still under debate and the sensitivity and specificity of different oral fluid assays and their relation to blood THC levels has been recently reviewed by Wennberg (50).

Our study has clear limitations. We studied the effects of different doses of cannabis but did not include alcohol. We asked drivers how confident they were in their ability to drive well before their performance and asked them to rate this on a scale of 1–100 (100 being best performance). Considerable interindividual variation was observed in evaluations of driving confidence, in reaction times, and in driving ability, which is a known problem in simulator studies. In an attempt to reduce interindividual variability, we did not include women as their simulator performance is known to differ from that of men and their higher body fat could affect the pharmacokinetics of cannabis. Our results thus only apply to men who nonetheless represent the majority of people found to be driving under the influence of drugs. The effects of interindividual variation were addressed by our statistical analysis which compared each individual performance across the three conditions. We excluded participants who were known to use multiple drugs and confirmed this at the start of each testing session using urine screening. We note that multiple drug use is frequent in the population and we cannot assess the effects of cannabis use in these cases. While we divided cannabis consumers into chronic and occasional consumers, we have no information on the strength of cannabis products they habitually which may influence the effects. However, the ability of cannabis consumers to self-titrate the amount of THC consumed may mitigate this effect. Finally, while all our occasional consumers completed the study, we had to overrecruit the chronic consumer group as four participants dropped out despite telephone reminders by the study team. Participants cited being unable to adhere to the study protocol, lack of motivation, or lack of organization as causes. It is unclear how this increased dropout of chronic consumers could affect our findings.

## 5 Conclusions

Cannabis consumption rapidly affects driving confidence and this normalizes slowly. Driving confidence is related to both THC concentrations and driving ability measured by the SDLP: unlike alcohol consumers, cannabis consumers have some insight into their impairment meaning they can potentially adopt countermeasures (e.g., lower speeds) to reduce accidents.

## Data availability statement

The raw data supporting the conclusions of this article will be made available by the authors, without undue reservation.

## Ethics statement

The studies involving humans were approved by Ethics Committee of Saint Germain en Laye. The studies were conducted in accordance with the local legislation and institutional requirements. The participants provided their written informed consent to participate in this study.

## Author contributions

JA was the scientific director and main designer of the study. IL and SH contributed to conception and design of the study. SH was the clinical investigator of the study, managed the recruitment, the inclusion of participants, and wrote the first draft of the manuscript. IL organized and controlled the study and was responsible for realization and data monitoring. NS and BC performed the statistical analysis. All authors contributed to manuscript revision, read, and approved the submitted version.
